# County-Level Trends in Cervical Cancer Incidence, Stage at Diagnosis, and Mortality in Kentucky

**DOI:** 10.1001/jamanetworkopen.2023.38333

**Published:** 2023-10-19

**Authors:** Haluk Damgacioglu, Todd Burus, Kalyani Sonawane, Elizabeth Hill, Krystle A. Lang Kuhs, Ashish A. Deshmukh

**Affiliations:** 1 Department of Public Health Sciences, Medical University of South Carolina, Charleston; 2 Hollings Cancer Center, Medical University of South Carolina, Charleston; 3 Markey Cancer Center, University of Kentucky, Lexington; 4 Department of Epidemiology & Environmental Health, College of Public Health, University of Kentucky, Lexington

## Abstract

This cross-sectional study evaluates recent trends in rates of cervical cancer incidence and incidence-based mortality among women in Appalachian and non-Appalachian Kentucky counties.

## Introduction

Kentucky had the highest incidence of cervical cancer of all US states from 2015 to 2019.^1^ The central Appalachian region in Kentucky faces high economic distress, persistent poverty, and health access inequity owing to isolation, lack of transportation, and health care shortage, contributing to suboptimal cervical cancer screening and care.^2,3,4^ Understanding county-level trends can elucidate the magnitude of disparities and inform public health interventions. In this cross-sectional study, we evaluated recent trends in cervical cancer incidence and incidence-based mortality (IBM) rates in Appalachian and non-Appalachian Kentucky counties.

## Methods

This study was deemed exempt from review and informed consent by the Medical University of South Carolina Institutional Review Board owing to use of deidentified patient data. We followed the STROBE reporting guideline.

We analyzed 2000 to 2019 Surveillance Epidemiology and End Results data for Kentucky. We identified microscopically confirmed malignant cervical cancer cases (*International Classification of Diseases for Oncology, Third Edition* site codes C53.0-C53.9 and histology codes 8010-8671 and 8940-8941).^5^ We calculated incidence rates by stage at diagnosis for the entire state and Appalachian (54 counties) and non-Appalachian (66 counties) regions and corrected them using population-level hysterectomy prevalence. We estimated hysterectomy-corrected cervical cancer IBM rates. We then calculated piecewise log-linear trends and annual percentage changes (APCs) and rate ratios (RRs) to examine incidence in Appalachian compared with non-Appalachian Kentucky and nationally (estimated using US cancer registry data) from 2017 to 2019.^5^ Statistical analysis was conducted from February 10 to April 17, 2023, using SEER*Stat, version 8.4.0.1, and Joinpoint Regression, version 4.8.0.1 (National Cancer Institute). Statistical significance was tested at 2-sided *P* < .05. Technical details are available in the eMethods in Supplement 1.

## Results

**Figure 1.**
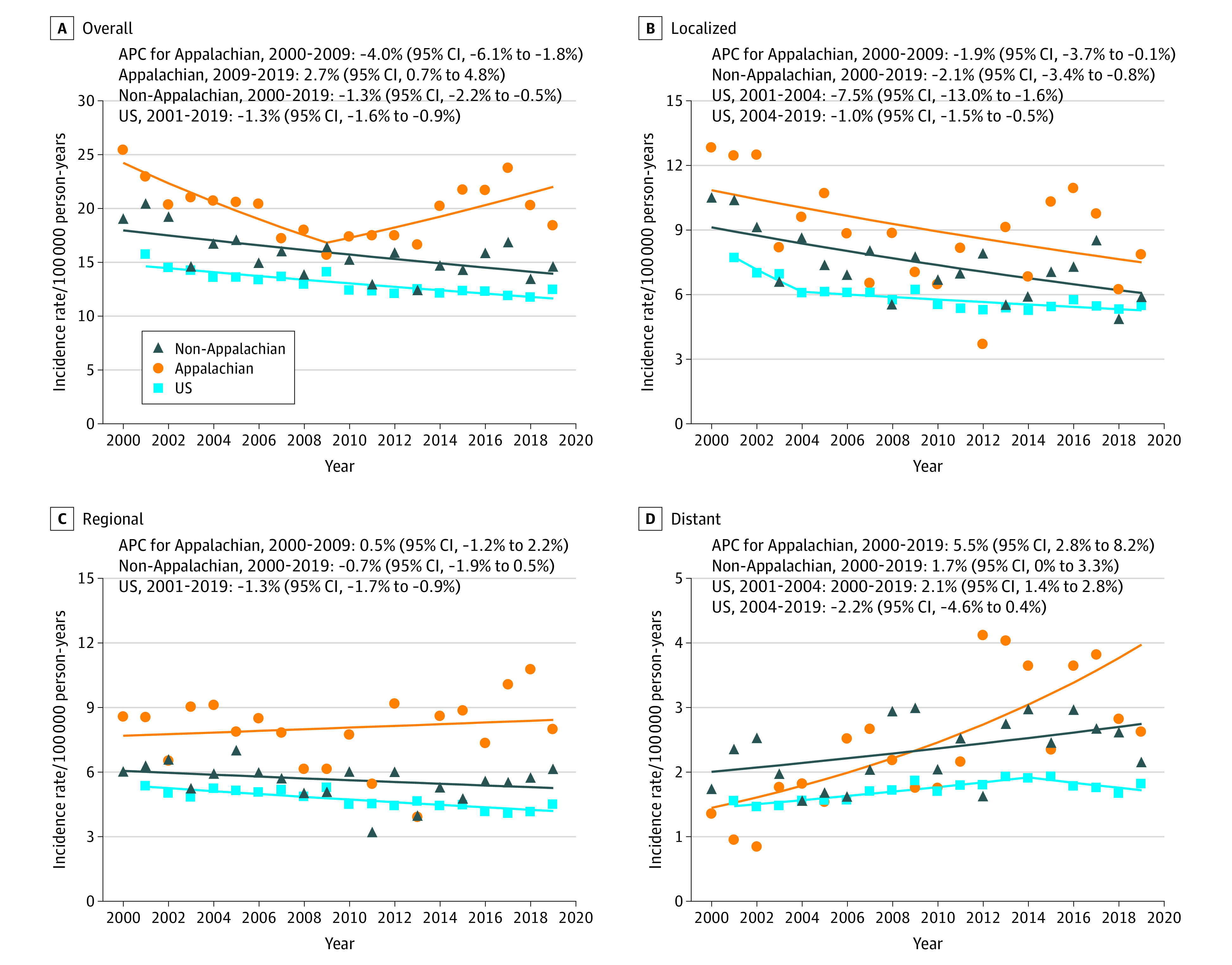
Trends in Hysterectomy Cervical Cancer Incidence Rates Overall and by Stage at Diagnosis Data are compared from Appalachian Kentucky, non-Appalachian Kentucky, and the entire US using the 2000-2019 Surveillance, Epidemiology, and End Results database. Incidence rates were estimated for women 15 years or older to reflect incidence accurately among age groups at risk of developing cervical cancer (as cervical cancer is rare among young women younger than 15 years: <3 cases in Kentucky and <16 cases nationally 2000-2019). Rates were calculated as number of cases per 100 000 person-years and were age adjusted to the 2000 US population. Small numbers (<10 cases/y in Kentucky) precluded characterization of trends in cervical cancer with unknown stage at diagnosis. APC indicates annual percentage change.

From January 1, 2000, to December 31, 2019, 4110 cervical cancer cases (1339 [32.6%] in Appalachia; 2781 [67.7%] in non-Appalachia; mean [SD] patient age, 50.0 [15.0] years) were reported in Kentucky. Race and ethnicity, abstracted from medical records, included 74 Hispanic (2.0%), 328 non-Hispanic Black (8.0%), 3648 non-Hispanic White (88.8%), and 60 non-Hispanic other (including American Indian or Alaska Native, Asian, Native Hawaiian or Other Pacific Islander, and unspecified) (1.5%) women. In Appalachian Kentucky, annual incidence increased 2.7% (95% CI, 0.7%-4.8%) from 2009 to 2019 after an initial decline (APC for 2000-2009, −4.0% [95% CI, −6.1% to −1.8%]) (Figure 1A); in non-Appalachia, it decreased (APC for 2000-2019, −1.3% [95% CI, −2.2% to −0.5%]). Trends by stage at diagnosis are described in Figure 1B to D. Incidence per 100 000 in Appalachian Kentucky (vs the US) was higher overall during 2017 to 2019 (20.7 vs 12.0; RR, 1.7 [95% CI, 0.5-2.0]) and for localized (8.0 vs 5.4; RR, 1.5 [95% CI, 1.2-1.8]), regional (9.6 vs 4.2; RR, 2.3 [95% CI, 1.8-2.7]), and distant (3.1 vs 1.8; RR, 1.8 [95% CI, 1.2-2.5]) stages.

**Figure 2.**
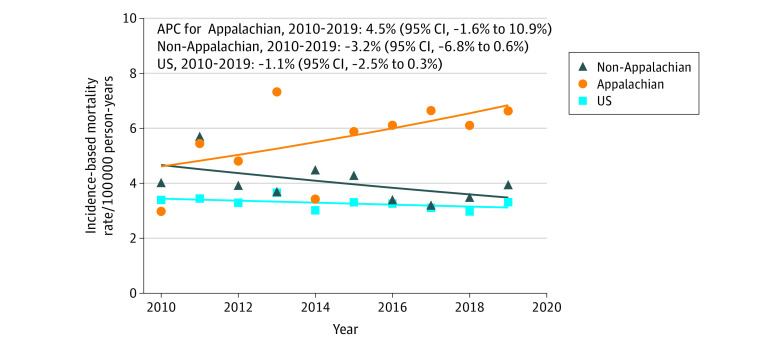
Trends in Hysterectomy-Corrected Cervical Cancer Incidence-Based Mortality Rates Data are compared from Appalachian Kentucky, non-Appalachian Kentucky, and the entire US using the 2010-2019 Surveillance, Epidemiology, and End Results database Incidence-based mortality rates were estimated for cervical cancer cases diagnosed during 2000 to 2019. Rates for cervical cancer incidence-based mortality were calculated as a number of deaths per 100 000 person-years and were age-adjusted to the 2000 US standard population. APC indicates annual percentage change.

From 2010 to 2019, there were 563 reported cervical cancer deaths: 191 (33.9%) in Appalachia (188 non-Hispanic White [98.4%]) and 372 (66.1%) in non-Appalachia (330 non-Hispanic White [88.7%]). The APC for cervical cancer IBM for Appalachian Kentucky was 4.5% (95% CI, −1.6% to 10.9%); for non-Appalachian Kentucky, it was −3.2% (95% CI, −6.8% to 0.6%) (Figure 2). During 2017 to 2019, cervical cancer mortality was 2-fold greater (6.4 vs 3.1 per 100 000; RR, 2.1 [95% CI, 1.6-2.6]) in Appalachian Kentucky than nationally.

## Discussion

Since 2009, cervical cancer incidence and mortality have increased dramatically in Appalachian Kentucky. Our findings suggest that the rise in cervical cancer incidence is not an artifact of increased screening^5^ and may reflect reduced access to screening and preventive care, greater disruptions along the screening-to-treatment continuum, use of less effective screening approaches, or increased prevalence of risk factors.

These findings could help generate hypotheses for future research and drive public health response. Study limitations include the unavailability of information regarding risk factors and screening and self-reported hysterectomy data that are subject to misclassification (although accuracy of self-report is comparable with medical records^6^). Future research and improvement in prevention is needed to combat magnifying disparities.

## Notes

Supplement 1. **eMethods.** Statistical Analysis eReferences

Supplement 2.
Data Sharing Statement
