# Supplementary material for: County-Level Trends in Cervical Cancer Incidence, Stage at Diagnosis, and Mortality in Kentucky
Source: JAMA Netw Open. 2023 Oct 19;6(10):e2338333. doi: 10.1001/jamanetworkopen.2023.38333 (PMC10587791; doi:10.1001/jamanetworkopen.2023.38333)
Supplement: Supplement 1. — eMethods. Statistical Analysis eReferences [file jamanetwopen-e2338333-s001.pdf]

Supplementary Online Content

Damgacioglu H, Burus T, Sonawane K, Hill E, Lang Kuhs K, Deshmukh AA. County-level trends in cervical cancer incidence, stage at diagnosis, and mortality in Kentucky. *JAMA Netw Open*. 2023;6(10):e2338333. doi:10.1001/jamanetworkopen.2023.38333

eMethods. Statistical Analysis

eReferences

This supplementary material has been provided by the authors to give readers additional information about their work.

© 2023 Damgacioglu H et al. *JAMA Network Open*.

eMethods. Statistical Analysis

Overview

We analyzed the 2000-2019 Surveillance Epidemiology and End Results (SEER) data (SEER-17). The database encompasses around 26.5% of the United States population and incorporates 17 population-based registries including the Kentucky Cancer Registry. We identified microscopically confirmed malignant cervical cancer cases among individuals coded as female residing in Kentucky using the International Classification of Diseases for Oncology, Third Edition site codes C53.0-C53.9, and histology codes 8010-8671/8940-8941 [1]. We excluded cases diagnosed by autopsy or death certificate only. Histology codes 8050-8084 were used to identify cervical squamous cell carcinomas (SCC). We calculated hysterectomy cervical cancer incidence rates (overall) and squamous cell carcinoma (SCC) by stage at diagnosis for the entire state and for Appalachian (54 counties) and non-Appalachian (66 counties) regions. Incidence rates were estimated for women aged ≥15 years to reflect incidence accurately among age groups at risk of developing cervical cancer (as cervical cancer is rare among young women aged <15 years: 0 cases in Kentucky and <16 cases nationally during 2000-2019). Cervical cancer incidence rates were corrected using population-level hysterectomy prevalence rates.

Hysterectomy-corrected incidence rates

Hysterectomy prevalence was estimated using data from the Behavioral Risk Factor Surveillance System (BRFSS) for each age group (18-24, 25-29, 30-34, 35-39, 40-44, 45-49, 50-54, 55-59, 60-64, 65-69, 70-74, 75-79, 80+). BRFSS is a nationally representative telephone survey that captures behavioral risk information at both state and local levels across the entire US population. To ensure consistent and reliable estimates of hysterectomy prevalence, our analysis was based on data collected from women residing in Kentucky ($N_{2001\text{-}2019}$=52,418). Utilizing logistic regression, we computed survey-weighted prevalence estimates, factoring in age groups, years, and an interaction term for age group and year using data from BRFSS. Adjusted prevalence rates were estimated from the model, within distinct age groups and year strata. These adjusted prevalence rates were then used to estimate the corresponding population at-risk by excluding women with a history of hysterectomy from the denominator. To appropriately account for instances where a hysterectomy was performed due to cervical cancer treatment, we added those cases back into the adjusted denominator. All rates corrected for hysterectomy prevalence were standardized to the U.S. population of 2000. Similar methods were used in several studies [2, 3].

Incidence-based mortality rates

Incidence-based mortality (IBM) measures the relationship between disease incidence and mortality rates. Cervical cancer mortality trends (estimated using information recorded in death certificate) has been considered as a measure of progress against cervical cancer in the United States (US). However, a limitation of this measure is that death certificates lack information regarding the onset of disease (e.g. age at diagnosis) and tumor histology (e.g., squamous cell carcinoma that is largely amenable to screening), limiting the ability to report calendar trend by these important tumor diagnostic characteristics. Different from observed mortality estimated using death certificate data, IBM focuses on deaths that occur after a diagnosis of a disease.

© 2023 Damgacioglu H et al. *JAMA Network Open*.

In our study, we analyzed cervical cancer IBM rates which provide insights into the mortality attributed to cervical cancer. We utilized the IBM file which links cervical cancer deaths to incident cervical cancer cases in the SEER18 database. However, the SEER18 file exclusively covers incident cases diagnosed from 2000 to 2019. Consequently, incidence-based mortality rates present an underestimation in earlier years due to insufficient follow-up duration for a reliable mortality estimate. Hence, our analysis concentrated on IBM rates within the 2010 to 2019 timeframe.

Trend analysis

We utilized joinpoint regression analysis to examine the temporal trends in cervical cancer incidence rates and incidence-based mortality rates. Joinpoint regression fits a series of joined straight lines on a logarithmic scale to depict the patterns within annual age-standardized rates. We employed the Joinpoint regression analysis software version 4.9.1.0, provided by the Surveillance Research Program of the US National Cancer Institute. The joinpoint regression analysis identified significant inflection points, called "joinpoints," in the cervical cancer rate trends. These joinpoints, determined using the calendar year as a regression variable, indicated substantial shifts in the trends. The analysis employed a heteroscedastic/uncorrelated error joinpoint model, factoring in standard errors for age-adjusted rates while assuming Poisson distributions for cancer counts. A Monte Carlo permutation test was conducted, involving 4499 randomly selected datasets to identify the optimal-fit line for each segment [4]. The analysis commenced with a baseline of zero joinpoints (representing a straight line) and iteratively assessed the significance of trend changes by progressively adding more joinpoints, up to a predetermined maximum [4]. The model incorporated a maximum of one joinpoint for the 2010 to 2019 incidence-based mortality trends, and up to three joinpoints for the incidence trends. To ascertain the significance of trends, a t-test was employed for zero joinpoints, while a z-test was used for one or more joinpoints. We considered statistical significance at a P value of less than 0.05, and all hypotheses were evaluated on a two-sided basis.

© 2023 Damgacioglu H et al. *JAMA Network Open*.

eReferences

1. Shahmoradi, Z., Damgacioglu, H., Clarke, M. A., Wentzensen, N., Montealegre, J., Sonawane, K., & Deshmukh, A. A. (2022). Cervical Cancer Incidence Among US Women, 2001-2019. JAMA, 328(22), 2267-2269.
2. Clarke, M. A., Devesa, S. S., Harvey, S. V., & Wentzensen, N. (2019). Hysterectomy-corrected uterine corpus cancer incidence trends and differences in relative survival reveal racial disparities and rising rates of nonendometrioid cancers. Journal of Clinical Oncology, 37(22), 1895.
3. Cohen, C. M., Wentzensen, N., Castle, P. E., Schiffman, M., Zuna, R., Arend, R. C., & Clarke, M. A. (2023). Racial and ethnic disparities in cervical Cancer incidence, survival, and mortality by histologic subtype. Journal of Clinical Oncology, 41(5), 1059-1068.
4. Kim HJ, Fay MP, Feuer EJ, Midthune DN. Permutation tests for joinpoint regression with applications to cancer rates. Stat Med 2000;19:335-51 (correction: 2001;20:655).

© 2023 Damgacioglu H et al. *JAMA Network Open*.
