# Supplementary material for: County-Level Trends in Cervical Cancer Incidence, Stage at Diagnosis, and Mortality in Kentucky
Source: JAMA Netw Open. 2023 Oct 19;6(10):e2338333. doi: 10.1001/jamanetworkopen.2023.38333 (PMC10587791; doi:10.1001/jamanetworkopen.2023.38333)
Supplement: Supplement 2. — Data Sharing Statement [file jamanetwopen-e2338333-s002.pdf]

Data Sharing Statement

Damgacioglu. County-Level Trends in Cervical Cancer Incidence, Stage at Diagnosis, and Mortality in Kentucky. *JAMA Netw Open*. Published October 19, 2023.
doi:10.1001/jamanetworkopen.2023.38333

Data
Data available: Yes
Data types: Deidentified participant data
How to access data: De-identified data are available publicly.
When available: With publication

Supporting Documents
Document types: None

Additional Information
Who can access the data: Anyone requesting the data
Types of analyses: for any purpose
Mechanisms of data availability: with investigator support
Any additional restrictions: Data are publicly available. NCI recommends to not include datapoints with <10 cancer cases.
